# Acute Cholecystitis Leading to Elevated High-Sensitive Cardiac Troponin I in a Young Female Without Any Cardiac Ailment: A Rare Case Presentation

**DOI:** 10.7759/cureus.33194

**Published:** 2022-12-31

**Authors:** Dnyanesh Amle, Nilaya Patil, Apurva Sakarde, Dr Jyoti John, Bhupendra Mehra

**Affiliations:** 1 Biochemistry, All India Institute of Medical Sciences, Nagpur, Nagpur, IND; 2 General Surgery, All India Institute of Medical Sciences, Nagpur, Nagpur, IND

**Keywords:** ck-mb, acute myocardial infarction, high sensitive cardiac troponin i, cardiac troponin i, acute cholecystitis

## Abstract

Cardiac troponin I (cTnI) is regarded as a gold standard investigation for the diagnosis of acute myocardial infarction (AMI). However, cTnI may be elevated in certain non-AMI cardiac conditions and even in certain noncardiac conditions.

We report a case of a young female presenting with symptoms suggestive of acute cholecystitis with elevated high-sensitive cardiac troponin I (hs-cTnI). The patient developed acute chest pain during the hospital stay. On evaluation, quantitative assay for hs-cTnI was found to be elevated; however, other markers of cardiac damage such as creatinine kinase-MB (CK-MB), qualitative cTnI by card test, and even echocardiogram (ECG) were found to be negative. As the patient was a young female with no significant history of coronary diseases, the spurious elevation of hs-TnI due to a noncardiac ailment was suspected. The patient was managed with minimal cardiological management till AMI was excluded. The hs-cTnI levels returned to normal post-cholecystectomy.

A patient presenting with symptoms suggestive of cholecystitis and elevated hs-cTnI must be carefully evaluated before resorting to any invasive management for AMI. In most cases, hs-cTnI will return to normal post-cholecystectomy.

## Introduction

Cardiac troponins, troponin T (cTnT), and troponin I (cTnI) are regarded as the gold standard for the diagnosis of acute myocardial infarction (AMI) [1]. Irrespective of etiopathogenesis, increased cTnI indicates reversible or irreversible myocardial damage with high specificity [2]. However, cTnT and cTnI are found to be elevated in certain non-AMI cardiac conditions such as pericarditis, endocarditis, myocarditis, cardiomyopathies, heart failure, cardiotoxicity of some therapeutic agents, and heart surgeries [3,4]. Also, certain noncardiac ailments have been associated with elevated troponins including heavy and prolonged physical activity, pulmonary embolism (PE), sepsis, acute and chronic kidney disease (CKD), and skeletal muscle damage [5,6]. Although acute cholecystitis is considered one of the differential diagnoses of AMI, it has rarely been reported to be associated with elevated cTnI and electrocardiographic (ECG) changes [7]. In this article, we report a case of elevated cTnI mimicking AMI in a young female with known acute cholecystitis.

## Case presentation

**Figure 1:**
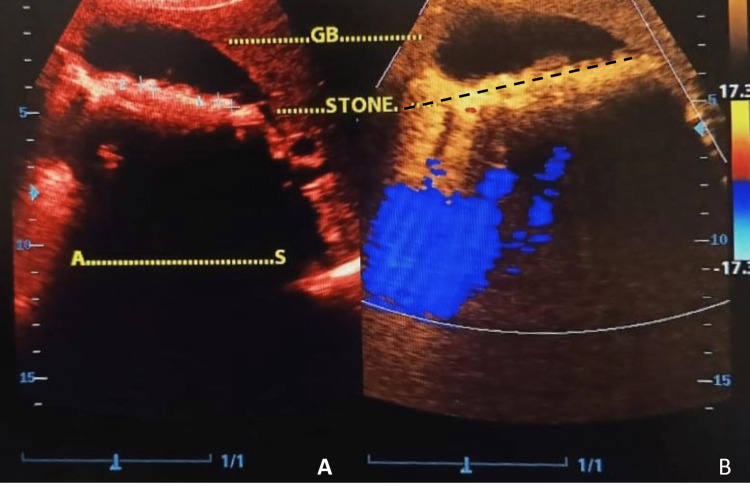
(A and B) Abdominal ultrasonography image (B-mode) showing distended gall bladder with stones, sludge, thickened gall bladder walls, and signs of cholecystitis GB: Gall bladder.

A 15-year-old female presented to the outpatient department with complaints of recurrent abdominal pain. The pain was of sudden onset, started in the right upper quadrant of the abdomen, and was associated with nausea. There was no history of vomiting, not associated with radiation or referral to other sites. There was no history of fever, trauma, urinary complaints, bowel or bladder habits alteration, sickle cell anemia, tuberculosis, or any associated menstrual complaints. On examination, the patient was normotensive, heart rate was 82/minutes, and there was no pallor, icterus, lymphadenopathy, cyanosis, clubbing, or edema. Epigastric tenderness was present with guarding and positive Murphy’s sign. On the clinical picture, acute cholecystitis was suspected. Abdominal and pelvic ultrasonography (USG) was ordered, and cholelithiasis with cholecystitis was reported in USG (Figure 1). The hematological evaluation including complete blood count (CBC) and hemoglobin electrophoresis was normal. Urine routine and coagulation assays were within normal limits. Liver function tests, renal function tests, and amylase levels were normal.

**Figure 2:**
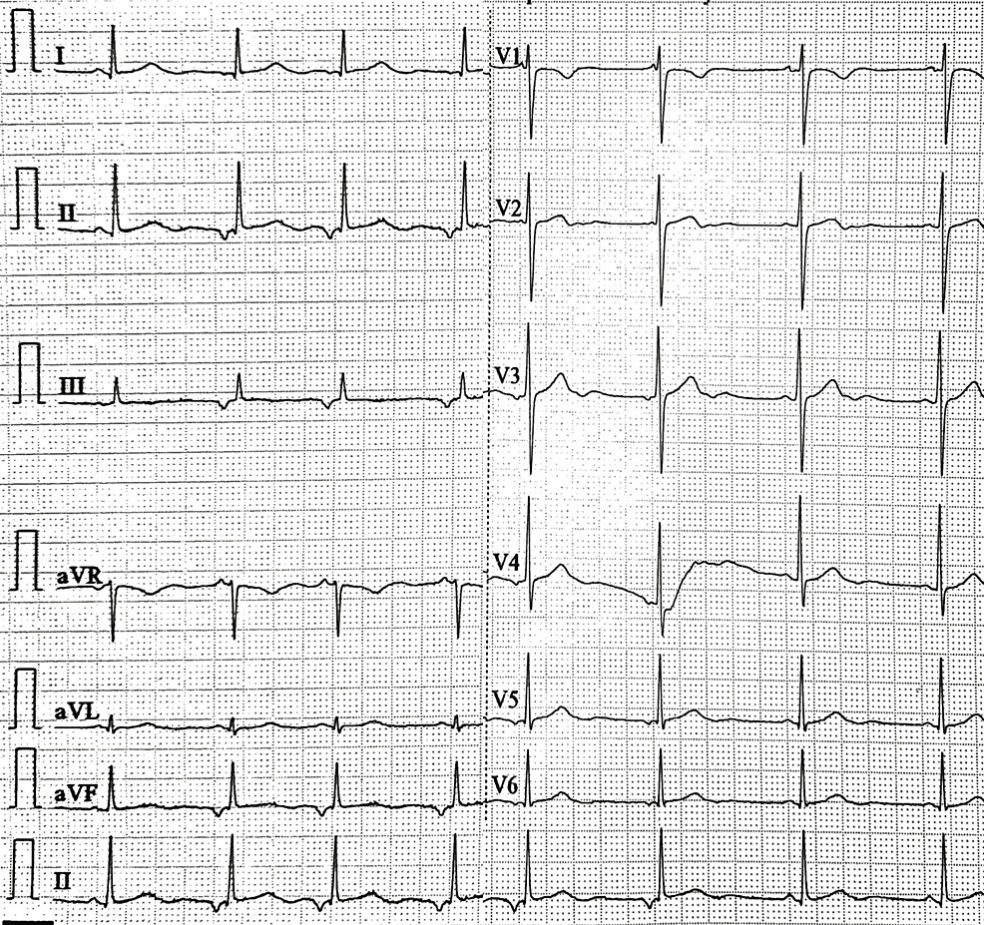
Electrocardiogram 12 lead strip showing findings not suggestive of AMI AMI: Acute myocardial infarction.

The patient complained of chest pain on the third day of admission. A cardiologist’s opinion was sought who requested troponin I (TnI) and ECG. The TnI was analyzed on VITROS® 5600 integrated system by Ortho Clinical Diagnostics, Johnson and Johnson^TM^, Rochester, NY using high-sensitive cardiac troponin I (hs-cTnI) assay kit (Cat. No.: 684 4436) based on the principle of enhanced chemiluminescence immunometric immunoassay as per the manufacturer’s instructions. The quality control was ensured by routine procedures of calibration (hs Troponin I Calibrator Pack 684 4437) checking followed by two levels of Liquid QC Cardiac Marker (“VS” controls Tri-level Cat. No.: 91474). The reported intraassay and interassay coefficient of variation (CV) were <10%. The hs-cTnI was found to be positive at 13 ng/L (internal reference: 9 ng/L). However, no findings suggestive of AMI were noted in ECG. Also, the ectopic auricular rhythm was noted on ECG (Figure 2). The test was repeated in the same sample as well as in another sample, keeping in consideration the young age and lack of risk factors for AMI. The results of high hs-cTnI were consistent.

**Figure 3:**
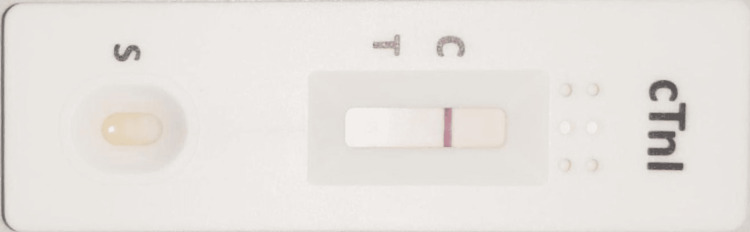
Qualitative cTnI test showing negative findings cTnI: Cardiac troponin I.

The cardiologist ordered a creatinine kinase-MB (CK-MB) quantitative assay, and a TnI qualitative analysis was also performed on the cTnI card test to confirm the findings of hs-cTnI. The patient was started on aspirin and unfractionated heparin (UFH) under the cardiologist's observation. CK-MB was performed on ADVIA® Centaur XPT (Siemense^TM^ Healthcare Diagnostic Inc, Tarrytown, NY) using CK-MB assay (Cat. No.: RA-67398) and was found to be 0.26 ng/ml (internal reference < 0.78 ng/ml). Also, card test using JusChek^TM^ cardiac troponin I rapid tests cassette by ACRO biotech Inc®. Rancho Cucamonga CA, USA (Cat. No.: Ref CTI-402) was found to be negative (Figure 3). The aspirin and UFH were withdrawn after these findings.

After the washout period of five days (confirmed by coagulation parameters), the patient was operated on by laparoscopic cholecystectomy. During surgery, a distended gall bladder with gallstones was found. The gall bladder was dissected and retrieved via a 10 mm port. The procedure was reported to be uneventful. The sample was sent for histopathology, which was reported to be in consistent with chronic cholecystitis with cholelithiasis. After follow-up on day 4 of surgery, the quantitative hs-cTnI level in the patient was found to be normal. The patient was discharged with oral antibiotics and analgesics.

## Discussion

We reported a case of elevated hs-TnI in a young patient presenting with cholecystitis. The CK-MB and qualitative cTnI were, however, found to be negative in the patient. Later findings were also consistent with the clinical history and ECG findings.

Acute cholecystitis and AMI sometimes overlap in presentation and clinical history [7]. Previously, the symptoms suggestive of AMI along with the pathognomonic finding of raised cTnT, cTnI, elevated CK-MB, and even elevated heart fatty acid binding (h-FABP) proteins have been observed in patients with acute cholecystitis [8-10]. These findings may or may not be associated with corresponding peculiar ECG changes such as ST-segment elevation, pathological Q wave, ST depression, and even inverted T wave [8,10,11]. The proposed mechanisms for elevated markers of cardiac ischemia are reduced coronary flow by the distended gall bladder and coronary vasospasm caused by vagal reflex (cardiobiliary reflex) [9,12,13]. Also, gall bladder distention may lead to elevated blood pressure and increased heart rate along with increased renin secretion [14]. The systemic inflammation associated with septicemia has also been claimed to be responsible for altered cTnI as well as ECG changes [15].

To the best of our knowledge, this is the youngest age to be reporting altered cardiac markers in cholecystitis. Previously, only a few studies have reported subjects with such findings without any ECG changes [16]. Also, ours is probably the first study to report these findings with hs-cTnI. Recently, hs-TnI has emerged as a reliable and highly sensitive method to diagnose myocardial damage. The lowest values of the limit of the blank (LoB), the limit of detection (LoD), the limit of quantitation (LoQ), and CV < 10% signify hs-cTnI as the most preferred investigation to diagnose AMI within minimal time after the event [1]. The non-AMI conditions in which hs-cTnI have been known to be elevated include increased arterial stiffness, left ventricular hypertrophy (mostly in male subjects), diabetes mellitus, cardiotoxic drugs, and statins [17]. However, most of these factors were ruled out in our patient on account of being a young, non-diabetic female, without any significant prior history of medication. Also, since hs-cTnI levels returned to normal after surgery, significant atherosclerosis as well as left ventricular hypertrophy are ruled out [17].

Thus, the elevated hs-TnI may have been caused by transient myocardial ischemia, which was minimal to even cause significant ECG changes, raise the CK-MB levels, or be detected by qualitative assay of cTnI.

## Conclusions

We conclude that a patient with acute cholecystitis might present with symptoms suggestive of AMI, which may be occasionally associated with elevated cTnI and CK-MB with or without ECG changes. The patient must be carefully evaluated for AMI before resorting to any invasive procedure. In most cases, cholecystectomy will lead to the normalization of markers of cardiac damage. These observations must always be kept in mind before taking any decision to carry out percutaneous coronary interventions or thrombolytic therapies in such cases.
